# Impact of a Nationwide Medication History Sharing Program on the Care Process and End-User Experience in a Tertiary Teaching Hospital: Cohort Study and Cross-Sectional Study

**DOI:** 10.2196/53079

**Published:** 2024-03-20

**Authors:** Jungwon Cho, Sooyoung Yoo, Eunkyung Euni Lee, Ho-Young Lee

**Affiliations:** 1 College of Pharmacy & Research Institute of Pharmaceutical Sciences, Seoul National University, Seoul, Republic of Korea; 2 Department of Pharmacy, Seoul National University Bundang Hospital, Gyeonggi-do, Republic of Korea; 3 Office of eHealth Research and Businesses, Seoul National University Bundang Hospital, Gyeonggi-do, Republic of Korea; 4 Department of Nuclear Medicine, Seoul National University College of Medicine, Seoul National University Bundang Hospital, Gyeonggi-do, Republic of Korea

**Keywords:** health information system, HIS, medication history, history, histories, patients’ own medication, satisfaction, DeLone and McLean Model of information systems success, value-based health care, emergency department, information system, information systems, emergency, urgent, drug, drugs, pharmacy, pharmacies, pharmacology, pharmacotherapy, pharmaceutic, pharmaceutics, pharmaceuticals, pharmaceutical, medication, medications, sharing, user experience, survey, surveys, intention, intent, experience, experiences, attitude, attitudes, opinion, perception, perceptions, perspective, perspectives, acceptance, adoption

## Abstract

**Background:**

Timely and comprehensive collection of a patient’s medication history in the emergency department (ED) is crucial for optimizing health care delivery. The implementation of a medication history sharing program, titled “Patient’s In-home Medications at a Glance,” in a tertiary teaching hospital aimed to efficiently collect and display nationwide medication histories for patients’ initial hospital visits.

**Objective:**

As an evaluation was necessary to provide a balanced picture of the program, we aimed to evaluate both care process outcomes and humanistic outcomes encompassing end-user experience of physicians and pharmacists.

**Methods:**

We conducted a cohort study and a cross-sectional study to evaluate both outcomes. To evaluate the care process, we measured the time from the first ED assessment to urgent percutaneous coronary intervention (PCI) initiation from electronic health records. To assess end-user experience, we developed a 22-item questionnaire using a 5-point Likert scale, including 5 domains: information quality, system quality, service quality, user satisfaction, and intention to reuse. This questionnaire was validated and distributed to physicians and pharmacists. The Mann-Whiteny *U* test was used to analyze the PCI initiation time, and structural equation modeling was used to assess factors affecting end-user experience.

**Results:**

The time from the first ED assessment to urgent PCI initiation at the ED was significantly decreased using the patient medication history program (mean rank 42.14 min vs 28.72 min; Mann-Whitney *U*=346; *P*=.03). A total of 112 physicians and pharmacists participated in the survey. Among the 5 domains, “intention to reuse” received the highest score (mean 4.77, SD 0.37), followed by “user satisfaction” (mean 4.56, SD 0.49), while “service quality” received the lowest score (mean 3.87, SD 0.79). “User satisfaction” was significantly associated with “information quality” and “intention to reuse.”

**Conclusions:**

Timely and complete retrieval using a medication history-sharing program led to an improved care process by expediting critical decision-making in the ED, thereby contributing to value-based health care delivery in a real-world setting. The experiences of end users, including physicians and pharmacists, indicated satisfaction with the program regarding information quality and their intention to reuse.

## Introduction

Health information systems (HISs) play a vital role in the delivery of health care services, as they provide access to the patient’s medical records, help track treatment progress, and support health care providers in making care decisions [1-3]. Although the development of HISs has revolutionized the provision of patient care and handling of patients’ health information, in the transitional period toward the era of the fourth industrial revolution, studies that evaluate humanistic outcomes as well as clinical or economic outcomes caused by HIS, are needed [4]. As leaders in health care settings have made various investments, such as time, money, and manpower, in managing HIS [5], the multifaceted evaluation of whether end users can use the HIS skillfully and achieve satisfaction in functionality and usability would be increasingly important in the future [4,6].

Health care organizations can ensure effective HIS use and improve the quality of patient care by conducting evaluations of HISs. These evaluations could allow health care organizations to proactively address issues related to system performance, integration, and data accuracy. However, evaluating the diversity and complexity of HISs in real-world clinical settings is a significant challenge [5,7]. Hospitals use different HISs depending on their work process, and the program related to direct patient care, including documentation and retrieval of medical records, or clinical decision support systems varies [8-10]. In addition, health care environments are constantly evolving with the emergence of innovative technologies [11]. Newly developed information systems or programs tend to be integrated into homegrown HISs after establishing a fully electronic medical record system. Thus, although HIS evaluations reporting economic, clinical, and humanistic outcomes could provide a balanced picture of the comprehensive impact of the health care interventions implemented, comprehensive evaluations of HISs are rarely conducted [12].

Acquisition of patients’ complete medication use history could greatly enhance medication management and support physicians in making informed decisions. Accurate and efficient compilation of information can be more important when time-sensitive clinical decisions and subsequent interventions are made [13], especially in the emergency department (ED). However, previous studies have demonstrated that accurate and timely collection of patients’ medication histories is challenging especially in the ED for various reasons, including patients with altered mental status due to confusion or intoxication, patients taking multiple outpatient prescriptions, and first-time patients to the hospital [14-16]. Since the treatment plan would change depending on the medication history, the prompt and complete evaluation of the medication history is vital. The process of collecting medication history was also described as a labor-intensive process, often requiring manual retrieval of information from outside the hospital [17,18]. Thus, a medication history sharing program called “Patient’s In-home Medications at a Glance” was developed and successfully launched within a homegrown HIS known as BESTCare in Seoul National University Bundang Hospital (SNUBH) on January 11, 2021. The program enabled health professionals to access the patients’ nationwide medication history swiftly and accurately from the Healthcare Insurance Review and Assessment Service database in South Korea with added features about the patient instructions and the identification guide for each medication. The rate of identification of patients’ medication history within 24 hours was significantly improved at the ED after the implementation of the program [19]. However, comprehensive evaluations of querying patient medication history were necessary to provide a balanced picture of the medication history program, as an HIS intervention could have had an impact not only on the care process but also on humanistic outcomes, such as end-user experience about its functionality and usability, which may evolve over time.

Therefore, this study aimed to evaluate the impact of an HIS intervention on health care delivery, namely medication history retrieval, using the “Patient’s In-home Medications at a Glance” program. Specifically, we evaluated the care process outcome, that is, the time from the first ED assessment to urgent percutaneous coronary intervention (PCI) initiation, and the humanistic outcome, that is, the end-user experience among physicians and pharmacists.

## Methods

### Study Design

**Figure 1.**
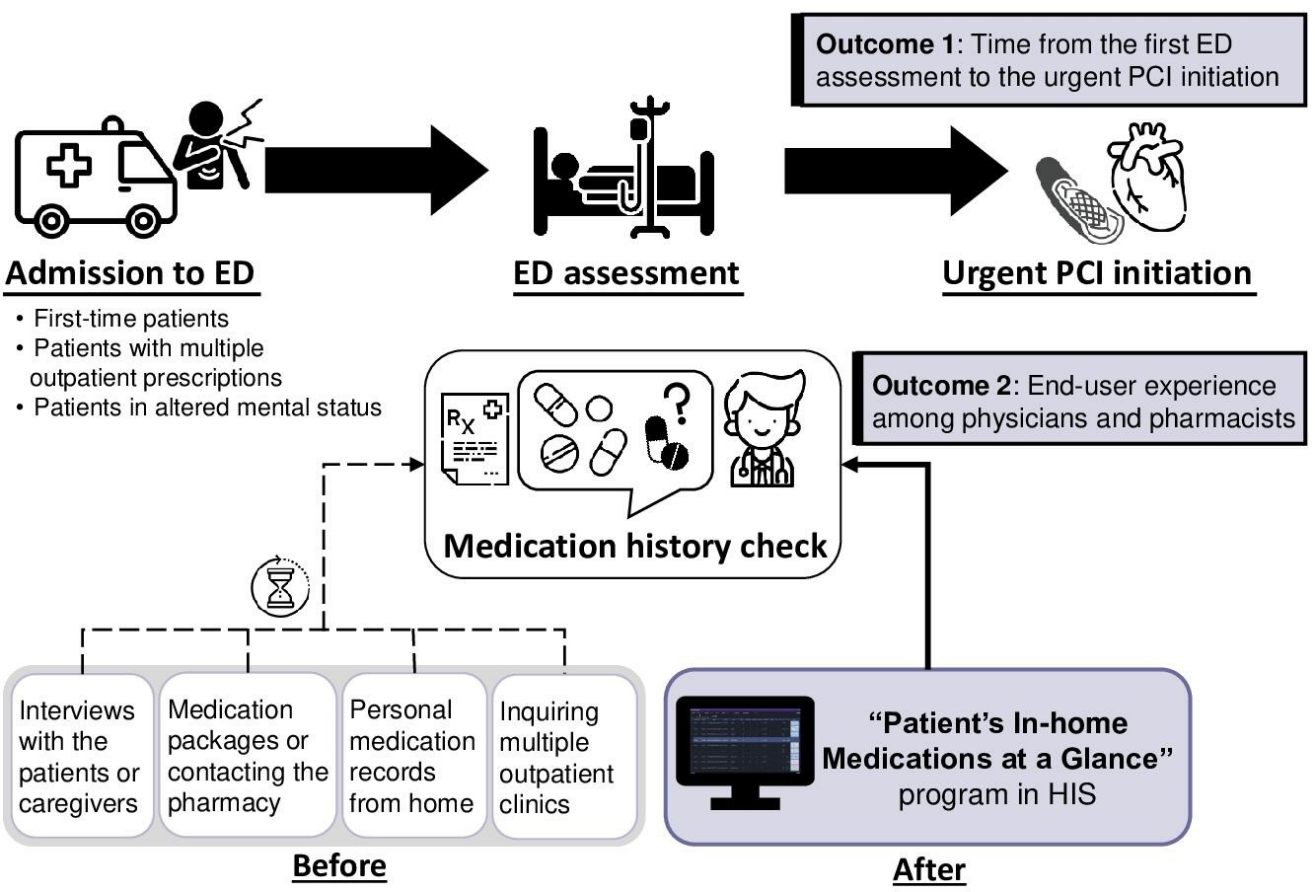
Emergency department (ED) process and medication history check depicting two outcomes: (1) time from the first ED assessment to urgent percutaneous coronary intervention (PCI) initiation as the care process outcome and (2) the end-user experience among physicians and pharmacists using the program as a humanistic outcome. Delayed medication history checks could increase the time of PCI initiation at the ED, especially in urgent clinical situations. The “Patient’s In-home Medications at a Glance” program linking to the nationwide personal medication records provides more rapid and complete collections of medication history compared to manual retrievals that often require interviews with patients or caregivers at the ED (icons are made by Freepik).

We conducted a cohort study and a cross-sectional study to evaluate both outcomes. We evaluated the impact of medication history retrieval using the “Patient’s In-home Medications at a Glance” program on two aspects: (1) the care process outcome and (2) the end-user experience among physicians and pharmacists. Figure 1 shows the ED process and medication history check to describe the 2 outcomes of this study.

First, we analyzed the care process to determine whether physicians’ use of the program could expedite the time from the first ED assessment to urgent PCI initiation. Second, to assess end-user experience, we developed a questionnaire consisting of 22 survey items that were validated. We then conducted a website-based survey among physicians and pharmacists who served as end users of the program.

### Care Process Outcome

#### Data Collection

For the care process, patients who were admitted to the ED for the first time from January 1, 2021, to December 31, 2022, were included to estimate the impact of the program on the collection of patients’ drug therapy. The outcome was defined as the time of initiating urgent PCI after the first assessment by ED physicians from January 1, 2021, to December 31, 2022. Urgent PCI was defined as PCI performed within an hour of admission to the ED. As the identification of the patient’s medication use history was required to further improve the care plan, the time from the first ED assessment to urgent PCI initiation was analyzed.

#### Data Analysis

To analyze the impact of the program on the care process, data were extracted from the SNUBH electronic database. We performed a Mann-Whitney *U* test to evaluate the difference in the time from the first ED assessment to urgent PCI initiation between patients who were queried about their medication use history by physicians via the program and those who were not.

All analyses were performed using IBM SPSS Statistics (version 22.0; IBM Corp) and R (version 4.0.2; R Foundation for Statistical Computing).

### Survey and Assessing Factors Affecting End-User Experience on the Program

#### Survey Development With a Conceptual Framework

**Figure 2.**
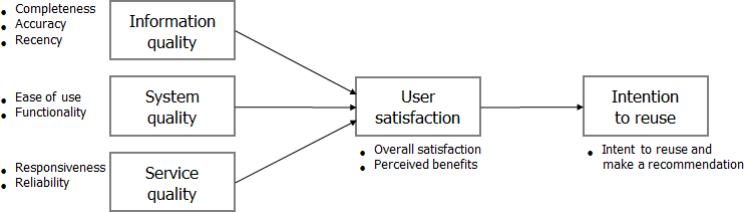
A research model for the survey development. The updated DeLone and McLean Model of Information Systems Success [20] provides domain variables consisting of 3 quality domains (ie, information, system, and service quality) as well as outcome domains.

To assess end-user experience and whether end users are satisfied with HIS and their intention to reuse it, we adopted the updated DeLone and McLean Model of Information Systems Success (DMISM) [20] for survey development. The updated DMISM provides a conceptual framework to suggest the factors necessary for the provision of use and benefits from the HIS. Based on the updated DMISM, we proposed that the quality of the information system consists of 3 quality domains: information quality, system quality, and service quality. These domains are necessary for user satisfaction and are instrumental in driving users’ intentions to reuse the system. In this study, we narrowed the scope to physicians and pharmacists who were already using the program. Therefore, we adjusted the factor of “intention to use” and “use” in the updated DMISM to “intention to reuse.” Due to the nature of the HIS, “intention to reuse” of the program by end users is considered the ultimate and crucial goal. By setting it as the final outcome variable, “intention to reuse” is influenced by preceding user satisfaction. Therefore, we established the research model with the relationship that “user satisfaction” affects “intention to reuse.” These domains were used to develop the survey (Figure 2).

We collected 32 survey questionnaires that assessed each quality domain regarding previous studies [3,21-24]. Through face validation with 6 pharmacists, a physician, and a medical informatics professor every 2 weeks for 3 months, the survey questionnaires were classified according to each domain. The questionnaires were eliminated or revised to reflect the contextual significance of the program. The draft survey finally consisted of 22 questionnaires, and a pilot study was conducted with 10 pharmacists and 2 physicians at SNUBH.

The survey was conducted from December 15, 2022, to December 28, 2022, at SNUBH. We used a web-based survey to collect data on the end-user experience efficiently and rapidly. The survey link was distributed to all physicians and pharmacists at the hospital via email. Survey completion was expected to take approximately 5 minutes. The items in the survey were rated on a 5-point Likert scale (1=not at all; 5=very much). Only those who provided consent after receiving an explanation of the background and purpose of the survey were included.

#### Data Analysis

An exploratory factor analysis of the results was then performed to determine how the items were classified into components. We used the Kaiser-Meyer-Olkin measure to assess sampling adequacy and obtained a specific value of 0.858, surpassing the recommended threshold of 0.5. The suitability of the data for factor analysis was further confirmed through the Bartlett test of sphericity, yielding a statistically significant result (*χ*^2^_105_=723.6; *P*<.001). The analysis of communality, indicating the explanatory power between measurement variables and extracted factors, was performed. Considering the general criterion that variables with communality below 0.4 are deemed low and should be excluded from factor analysis, 8 questions were excluded. Consequently, 14 questionnaires were retained (Table S1 in Multimedia Appendix 1).

Subsequently, we conducted a reliability analysis of the survey items and calculated Cronbach α. We analyzed the convergent and discriminant validity of the constructs. We used SPSS to conduct statistical analyses, including factor and reliability analyses. Finally, structural equation modeling (SEM) was used to evaluate the structural correlations among the domains using the AMOS 25 software (version 25.0; IBM Corp). SEM was chosen to provide a comprehensive understanding of the relationships among survey variables and to help validate the theoretical models with a visual representation.

### Ethical Considerations

This study was approved by the Institutional Review Board of SNUBH (B-2203-746-001; April 21, 2022), and the requirement of obtaining written consent was waived, as this study did not contain sensitive personally identifiable information.

## Results

### Care Process Outcome

Of the 162 patients who were admitted to the ED and visited the hospital for the first time over a 2-year period, 77 who underwent urgent PCIs within an hour from the first ED assessment to urgent PCI initiation were included. Patients who were regularly visiting hospitals with chronic diseases were excluded. Table 1 describes the demographic characteristics of patients, including gender, age, department, tests, and diagnosis, between the patient group (n=59), for which the doctor did not use the program, and the patient group (n=18), whose medications were accessed through the program.

**Table 1.** Demographics of patients receiving urgent percutaneous coronary intervention by use of the medication history program during the study period at an emergency department (n=77^a^).

Characteristics		No (without the program; n=59)	Yes (with the program; n=18)
Sex (male), n (%)		50 (84.7)	11 (61.1)
Age (years), mean (SD)		64.3 (12.1)	68.9 (12.4)
**Department at discharge, n (%)**			
	Cardiology	54 (91.5)	16 (88.9)
	Others	5 (8.5)	2 (11.1)
Had CT^b^ scan, n (%)		12 (20.3)	3 (16.7)
**Diagnosis, n (%)**			
	ST elevation myocardial infarction	53 (89.8)	16 (88.9)
	Others	10 (16.9)	4 (22.2)

a Patients receiving percutaneous coronary intervention within an hour at an emergency department from January 12, 2021, to December 31, 2022.

b CT: computed tomography.

Changes in time from the first ED assessment to urgent PCI initiation significantly decreased in patients who used the program (n=18; mean rank 28.72 min) versus patients who did not use the program (n=59; mean rank 42.14 min; Mann-Whitney *U*=346; *P*=.03).

### Survey and Assessing Factors Affecting End-User Experience on the Program

#### Survey Participants’ Characteristics

During the 2-week survey period, we received survey responses from 112 participants in the hospital. Among them, we removed the responses of 10 participants who never used the “Patient’s In-home Medication at a Glance” based on their answers to the first question. In addition, the responses of 5 participants who gave the same rating to the negative and positive questions were removed, as they were considered either not meaningful or not sincere to the survey, leaving 97 responses for analysis. Table 2 presents the characteristics. Participants included 62 (63.9%) physicians and 35 (36.1%) pharmacists, and the mean use count during the week was approximately 10.8 (SD 13.9).

**Table 2.** Participants’ characteristics (N=97).

Characteristics				Values, n (%)
**Occupation**				
	**Physician (n=62, 63.9%)**			
		**Position**		
			Professor	35 (56.5)
			Resident	27 (43.5)
		**Department**		
			Internal medicine	50 (80.6)
			Surgery	12 (19.3)
		**Workplace**		
			Ambulatory clinic	24 (38.7)
			General ward	21 (33.9)
			Emergency room	11 (17.7)
			Intensive care unit	6 (9.7)
	Pharmacist			35 (36.1)
**EHR**^**a**^ **experience (years)**				
	1			5 (5.2)
	3			20 (20.6)
	5			22 (22.7)
	10			20 (20.6)
	>10			30 (30.9)
**Sex**				
	Male			32 (33.0)
	Female			65 (67.0)
**Age (years)**				
	≤30			14 (14.4)
	31-40			58 (59.8)
	41-50			20 (20.6)
	>50			5 (5.2)
**Weekly frequency of using the program**				
	Mean (SD)			10.7 (13.9)
	Median (IQR)			6 (4-10)

a EHR: electronic health record.

#### Evaluation of the Survey Results

Of the 22 survey questions, the updated DMISM comprised 14 questions in 5 domains. After performing exploratory factor analysis, we calculated the mean score of each domain and Cronbach α to confirm the consistency of the items. This reliability analysis revealed that Cronbach α for all variables exceeded 0.80 (information quality: 0.808; system quality: 0.834; and service quality: 0.800), except for user satisfaction (Cronbach α=0.788) and intention to use (Cronbach α=0.795).

On a 5-point scale, the mean scores values for the information, system, and service quality of the program were 4.11 (SD 0.76), 4.24 (SD 0.75), and 3.87 (SD 0.79), respectively. User satisfaction (4.56, SD 0.49) and intention to reuse (4.77, SD 0.37) were measured. Among the 5 domains of the survey questionnaire, intention to reuse obtained the highest score. The estimates and weights of all 5 domains were analyzed, and no issues were observed in the convergent validity of the constructs (Table S2 in Multimedia Appendix 1). In addition, the subsequent analysis revealed the absence of discriminant validity (Table S3 in Multimedia Appendix 1).

#### Structural Correlations Between Domains

**Figure 3.**
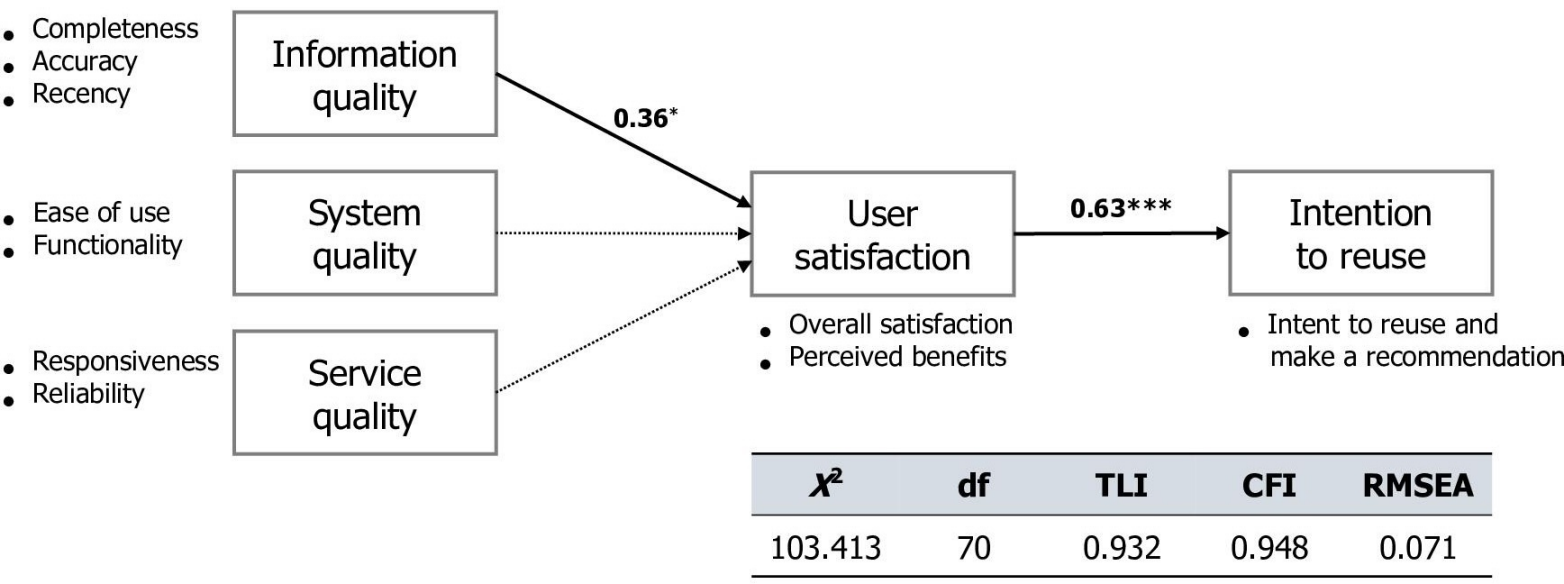
Results of the research model using the structural equation modeling analysis. Significant paths are indicated with solid lines, while nonsignificant paths are shown with dotted lines. Two significant paths are shown: information quality toward user satisfaction and user satisfaction toward intention to reuse. The standardized beta values are presented. *P=.01; ***P<.001. CFI: Comparative Fit Index; RMSEA: root mean square error of approximation; TLI: Tucker-Lewis Index.

The SEM images are shown in Figure 3. The model fit indices were calculated as follows: *χ*^2^_70_=103.413 (*P<.*001); goodness-of-fit index=0.868 (recommended: 0-1.0); root mean square residual=0.039 (recommended: 0-0.05); and root mean square error of approximation=0.071 (recommended: 0.05-0.08). The comparative fit index and the Tucker-Lewis index for the model exceeded 0.9. The normed fit index and adjusted goodness-of-fit index values were lower than the recommended values of 0.859 and 0.802, respectively. Thus, this model was confirmed to be appropriate for assessing the factors affecting the “intention to reuse” program as an end-user experience.

The associations between the latent variables were positive, supporting our hypotheses. Among the 3 quality domains, “information quality” had a significantly positive influence on “user satisfaction.” Consequently, the influence of “information quality” in “user satisfaction” and the influence of “user satisfaction” in “intent to reuse” were significantly associated.

## Discussion

### Principal Results

This study aimed to evaluate the impact of medication history retrieval using the “Patient’s In-home Medications at a Glance” program in homegrown HISs during the 2-year maintenance phase after program implementation. The significance of our findings was twofold. First, we conducted a comprehensive evaluation of the impact of the nationwide medication history-sharing program, consisting of care process outcomes and end-user experiences as humanistic outcomes. We elaborately planned both the care process and humanistic outcomes of 2-year use, which allowed the program to stabilize, after its implementation in the HIS [23]. The care process, focusing on the time required for urgent PCI initiation, was improved in the patient group, whose physicians used the program and experienced expedited urgent PCI initiation. Thus, the use of the program could help identify whether patients are taking an antiplatelet or anticoagulant agent when they are unconscious or are unable to identify their medications. Regarding humanistic outcomes, the survey showed high scores overall, especially for “user satisfaction” and “intention to reuse.” The increasing trend in the use of the “Patient’s In-home Medications at a Glance*”* program by physicians and pharmacists indicates the successful integration of the newly developed program into the HIS, as evidenced by a positive end-user experience.

Second, we assessed factors affecting end-user experience using SEM; “information quality” significantly influenced “user satisfaction,” and “user satisfaction,” in turn, positively enhanced “intention to reuse.” Since the survey was developed with the updated DMISM, which is a conceptual framework to suggest factors necessary for the “intention to reuse” the program, we could examine whether and how the 3 quality domains, including information, system, and service, affect “user satisfaction” and how “user satisfaction” affects “intention to reuse.” These findings highlight the potential of the HIS in supporting clinical decision-making and contributing to value-based health care through the provision of a comprehensive medication use history.

### Implications

Value-based health care is an approach to health care delivery in which providers are paid based on the patient’s health outcomes [25], while reducing costs [26]. The benefits of a value-based health care system include reduced treatment costs, increased care efficiency, and reduced risks [27]. Measuring a patient’s clinical outcomes is a major aim of value-based health care. In our study, we measured both care process outcomes and end-user experiences, which help present humanistic outcomes. Hence, a comprehensive evaluation was conducted by selecting both outcomes to determine the impact of the interventions using the HIS. Health service providers should provide patient-centered team care, share patients’ medical information, and measure the care process using the HIS. The physicians were able to collect the patients’ complete medication use histories in a friendly manner, even if the patients were unable to identify the exact medications they were taking. As access to a complete medication use history could help physicians make clinical decisions and collaborate care within the hospital [28], the HIS could help improve the patient’s outcomes. Thus, HISs can play a vital role in value-based health care by delivering comprehensive and up-to-date information, including medication use history, laboratory results, and other medical records.

In terms of the association between the survey domains, the updated DMISM was applied to identify the quality factors that contribute to “user satisfaction,” which affects end users’ “intention to reuse.” According to Alzahrani et al [29], 3 quality domains are significantly related to “user satisfaction” and “intention to reuse” and consequently affect actual usage. By conducting an SEM analysis of the survey results, our model revealed a significant effect of “information quality” on “user satisfaction,” as well as “user satisfaction” on “intention to reuse.” These results indicate that providing complete, accurate, and regent information is important for “user satisfaction,” ultimately driving the “intention to reuse.” A previous study stated that studies assessing the acceptance of HISs have been conducted from the physicians’ perspective, not the clinical pharmacists’ [30]. Since the program has been used by physicians and pharmacists, we could assess the factors affecting end-user experience in both professional groups. If the quality of information in an HIS is not guaranteed, health care professionals will not use specific programs in the HIS.

### Limitations

This study had some limitations. First, we developed and implemented the “Patient’s In-home Medications at a Glance” program in a single hospital. Thus, outcomes, such as care processes or factors affecting end-user experience, cannot be generalized to other hospitals in South Korea. However, as the Healthcare Insurance Review and Assessment Service has established guidelines for program development, further studies that use similar HISs could be conducted in other hospitals. Second, the pretest and posttest studies had the inherent limitations of nonrandomized, uncontrolled study designs. Although we showed the impact of the program on the time to PCI as the care process, we could not capture the long-term effects on clinical outcomes, such as survival rates or extended hospital stays. Nevertheless, our findings regarding the care process, specifically the reduction in time from the first ED assessment to urgent PCI initiation, could be meaningful not only in expediting clinical decisions but also in the evaluation of HISs in a real-world health care setting. Third, a notable limitation of our study is the imbalanced distribution of participants between the patient groups with or without the program (18 vs 59 participants) and the small number of patients in the group using the program. This uneven and small sample size raises concerns about the statistical robustness of our findings. Future research endeavors should prioritize achieving a more equitable number and distribution of patients to enhance the reliability and generalizability of our conclusions. Although our study offers valuable insights, the limitation of uneven and small sample sizes underscores the importance of cautious interpretation and highlights a potential area for improvement in subsequent research. Fourth, in the results of the SEM analysis, “information quality” was a standalone significant factor among 3 quality domains influencing “user satisfaction.” It is possible that the developed survey item may not adequately address the measurement of the quality domain. Lastly, our focus in this study was on system acceptability rather than the direct improvement in the health of the patients. We plan to focus more on the clinical outcome of the program, which includes not only medication information but also ensuring comprehensive disease management. This approach should be followed up for future measurements in subsequent studies.

### Conclusions

Our findings highlight the impact of the rapid and complete medication history retrieval using the “Patient’s In-home Medications at a Glance” program on the care process and end-user experience. A significantly positive effect was found on the care process by expediting urgent PCI initiation time at the ED, thereby contributing to value-based healthcare delivery in a real-world setting. Moreover, the HIS intervention provided high-quality information to physicians and pharmacists, resulting in high satisfaction. Long-term assessments can provide valuable insights into the sustained impact of the program, further optimizing patient outcomes.

## Supplementary material

10.2196/53079 Multimedia Appendix 1 Survey items, convergent validity, and discriminant validity.

## Abbreviations

DMISM: DeLone and McLean Model of Information Systems Success
ED: emergency department
HIS: health information system
PCI: percutaneous coronary intervention
SEM: structural equation modeling
SNUBH: Seoul National University Bundang Hospital
