# Supplementary material for: Impact of a Nationwide Medication History Sharing Program on the Care Process and End-User Experience in a Tertiary Teaching Hospital: Cohort Study and Cross-Sectional Study
Source: JMIR Med Inform. 2024 Mar 20;12:e53079. doi: 10.2196/53079 (PMC11004625; doi:10.2196/53079)
Supplement: Multimedia Appendix 1 [file medinform-v12-e53079-s001.docx]

**Table S1.** Survey items.

| **Questionnaire** |
| --- |
| **Information quality**  The information in the “*Patient’s In-home Medications at a Glance*” program is accurate.  The information in the “*Patient’s In-home Medications at a Glance*” program is always updated.  The “*Patient’s In-home Medications at a Glance*” program includes all medications that the patient used at home. |
| **System quality**  The “*Patient’s In-home Medications at a Glance*” program is easy to use.  The layout of the “*Patient’s In-home Medications at a Glance*” program is clear and concise. |
| **Service quality**  The “*Patient’s In-home Medications at a Glance*” program is flexible enough to interact with other health information systems.  There is adequate technical support from the system’s provider.  The *Help* function in the “*Patient’s In-home Medications at a Glance*” program is useful. |
| **User satisfaction**  I am satisfied using the “*Patient’s In-home Medications at a Glance*” program.  The “*Patient’s In-home Medications at a Glance*” program helps improve clinical decision-making process.  The “*Patient’s In-home Medications at a Glance*” program helps improve efficiency in providing patient care. |
| **Intention to reuse**  Using the “*Patient’s In-home Medications at a Glance*” program has improved my job performance in checking patients’ medications at home.  I am willing to use the “*Patient’s In-home Medications at a Glance*” program in checking patients’ medications at home again.  I am willing to recommend the “*Patient’s In-home Medications at a Glance*” program to other staff. |

**Table S2.** Convergent validity.

| **Construct** | **Item** | **Estimates** | | **S.E.** | **C.R.** | **AVE**  **(>0.5)** | **C.R.**  **(>0.7)** |
| --- | --- | --- | --- | --- | --- | --- | --- |
|  |  | **B** | **ß** |  |  |  |  |
| Information quality | IQ1 | 1 | 0.78 |  |  | 0.657 | 0.852 |
|  | IQ2 | 0.941 | 0.807 | 0.125 | 7.5 |  |  |
|  | IQ3 | 0.648 | 0.733 | 0.094 | 6.899 |  |  |
| System quality | SyQ1 | 1 | 0.84 |  |  | 0.793 | 0.885 |
|  | SyQ2 | 0.979 | 0.853 | 0.114 | 8.585 |  |  |
| Service quality | SeQ1 | 1 | 0.915 |  |  | 0.631 | 0.835 |
|  | SeQ2 | 1.171 | 0.709 | 0.149 | 7.845 |  |  |
|  | SeQ3 | 1.02 | 0.725 | 0.126 | 8.093 |  |  |
| User satisfaction | US1 | 1 | 0.817 |  |  | 0.802 | 0.923 |
|  | US2 | 0.866 | 0.806 | 0.116 | 7.482 |  |  |
|  | US3 | 0.726 | 0.641 | 0.119 | 6.085 |  |  |
| Intent to reuse | IU1 | 1 | 0.908 |  |  | 0.896 | 0.962 |
|  | IU2 | 0.509 | 0.715 | 0.071 | 7.135 |  |  |
|  | IU3 | 0.524 | 0.649 | 0.081 | 6.441 |  |  |

AVE: average variance extracted, C.R.: construct reliability, S.E.: standard error

**Table S3.** Discriminant validity.

| **Construct** | **1** | **2** | **3** | **4** | **5** | **AVE** | **C.R.** |
| --- | --- | --- | --- | --- | --- | --- | --- |
| 1 Information quality | 1 |  |  |  |  | 0.657 | 0.852 |
| 2 System quality | 0.332 | 1 |  |  |  | 0.793 | 0.885 |
| 3 Service quality | 0.370 | 0.635 | 1 |  |  | 0.631 | 0.835 |
| 4 User satisfaction | 0.316 | 0.346 | 0.346 | 1 |  | 0.802 | 0.923 |
| 5 Intent to reuse | 0.413** | 0.225 | 0.194 | 0.314 | 1 | 0.896 | 0.962 |

All correlation coefficients between factors were significant at *P*-value<.001, except **, with *P*-value=.001.
